# Congenital Cervicofacial Haemangioma With Deep Airway Extension

**DOI:** 10.7759/cureus.82276

**Published:** 2025-04-14

**Authors:** Mohamed Elmarghani, Fergus Cooper, Irfaan Mohangee, Mecaelan Sardar, Zaahir Cassimjee

**Affiliations:** 1 Otolaryngology, Aberdeen Royal Infirmary, Aberdeen, GBR; 2 Oncology, Palliative Care, Western General Hospital, Edinburgh, GBR; 3 Internal Medicine, Medway Maritime Hospital, Gillingham, GBR; 4 General Surgery, Mediclinic, Ermelo, ZAF

**Keywords:** congenital haemangioma, head and neck anaesthesia, head and neck pathologies, laryngeal hemangioma, obstructive airway diseases

## Abstract

The authors present a compelling case of a man in his 50s whose long-standing congenital cervical haemangioma became symptomatic after adopting a more physically active lifestyle. Imaging revealed notable findings, with ENT assessment uncovering deep airway extension of the hemangioma, triggering neck discomfort after strenuous activity. The patient had previously been advised to avoid general anaesthesia due to anticipated difficulties with intubation. Subsequent flexible laryngoscopy and CT imaging revealed a greater degree of airway involvement than initially suspected. The article explores the range of management options available for this condition. Remarkably, despite the potential complications, the patient continues to enjoy a good quality of life without requiring medical or surgical intervention.

## Introduction

Congenital haemangiomas (CHs) are rare, benign vascular tumours that develop in utero and are fully formed at birth. Unlike the more common infantile haemangiomas, which typically exhibit a proliferative phase during the first year of life before undergoing involution, CHs are distinct in that they are present at birth and follow different biological trajectories. They are classified into three subtypes: rapidly involuting CH (RICH), which regress spontaneously within the first year of life; non-involuting CH (NICH), which persist and continue to grow proportionally with the child; and partially involuting CH (PICH), which undergo partial regression before stabilising [1].

Although CHs are uncommon, they are clinically significant due to their potential to cause complications, particularly when located in critical anatomical regions, such as the head and neck. A retrospective cohort study conducted in France, which analysed data from 57 patients over a 13-year period, found that 33% of all CHs occurred in the head and neck region, second only to the limbs, which accounted for 55% of cases [1].

The clinical presentation of CHs varies considerably, ranging from small, isolated lesions causing a cosmetic burden to larger tumours extending into the airway, leading to functional impairment, such as voice changes and exercise intolerance [2]. Managing CHs poses significant challenges due to the unpredictability of their growth patterns and their potential for complications. While surgical excision is an effective treatment option in many cases, its feasibility is often dependent on tumour size and anatomical location, particularly when vital structures such as the airway, nerves, or major blood vessels are involved [3].

This case report examines an adult patient with a symptomatic cervicofacial haemangioma extending into the airway, presenting a unique clinical challenge. The case is particularly noteworthy given the rarity of CHs in adults and the involvement of the airway, which complicates both diagnosis and management. The unusual nature of this case highlights the importance of considering CH in the differential diagnosis of vascular lesions in the head and neck, even in adulthood. Furthermore, it underscores the need for a multidisciplinary approach to optimise management. This report will explore the diagnostic process, imaging modalities, treatment options, and follow-up strategies to provide a comprehensive understanding of how these rare lesions can be effectively managed in clinical practice.

## Case presentation

A 57-year-old gentleman was assessed in the head and neck clinic for a congenital left-sided haemangioma affecting the head and neck, which had recently become symptomatic.

Approximately one month prior, he had adopted a more active lifestyle, engaging in running and gym-based exercise with the aim of losing weight. However, during physical activity, he experienced neck discomfort and occasional minor bleeding from the cutaneous aspect of the haemangioma on his left ear.

He denied symptoms of dysphagia, odynophagia, dysphonia, haemoptysis, haematemesis, or stridor. There had been no noticeable changes in the size or discolouration of the lesion externally, nor had he experienced any other significant systemic symptoms.

On examination, a raised, bumpy, purplish lesion extended from his left ear down the face and across the neck, terminating at the midline and at the level of the left clavicle. There were associated soft tissue changes and swelling deep to the lesion. Unfortunately, images of skin lesions were not available.

Otoscopic examination was normal bilaterally. The oral cavity appeared unremarkable, and neck movements were unrestricted.

**Figure 1:**
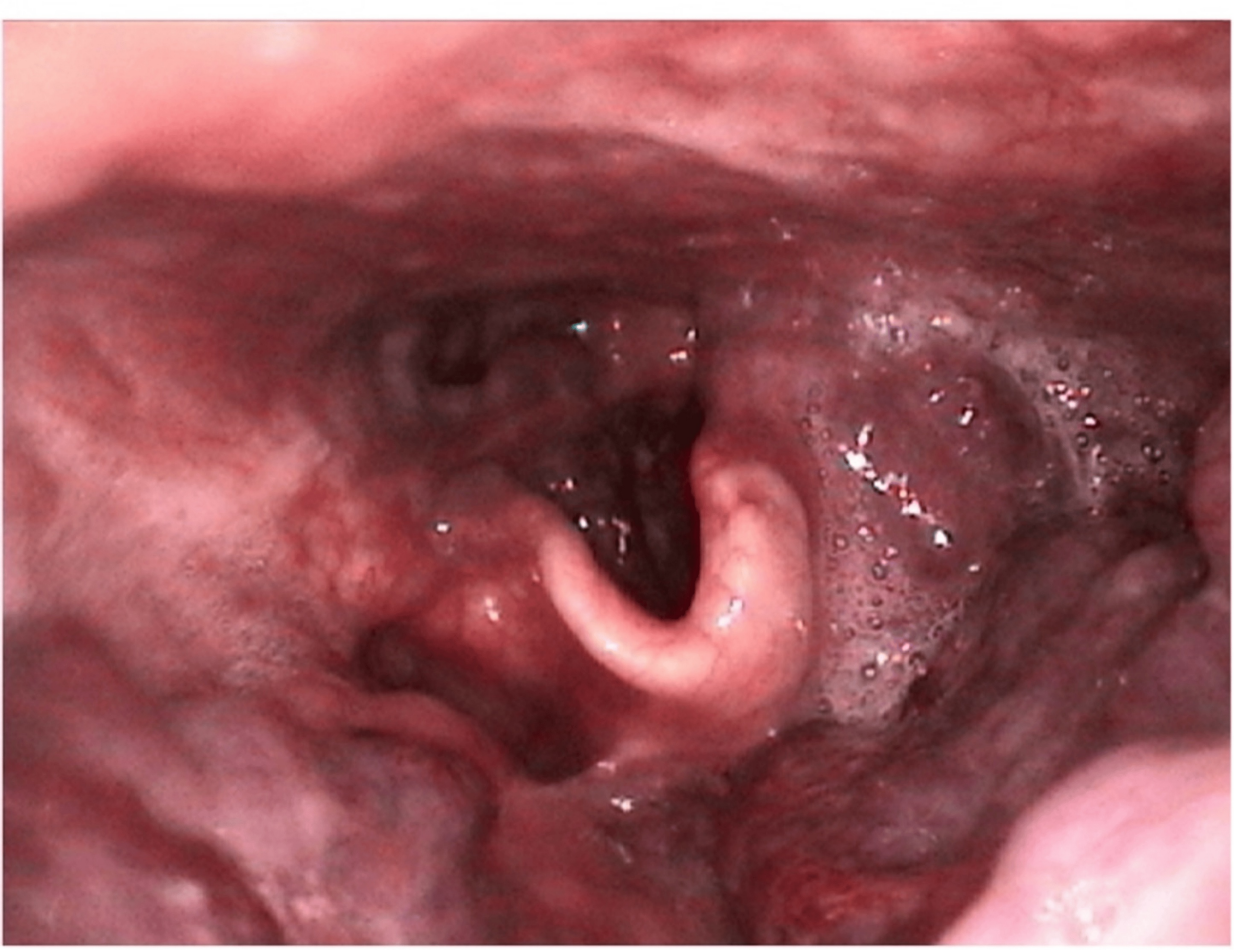
Supraglottic view of a flexible nasopharyngolaryngoscopy depicting encroachment of the haemangioma on the left side of the oropharynx

Flexible nasopharyngolaryngoscopy revealed mass involvement of the right tongue base, left anterior tonsillar pillar, and the left supraglottis and vallecula (Figure 1).

**Figure 2:**
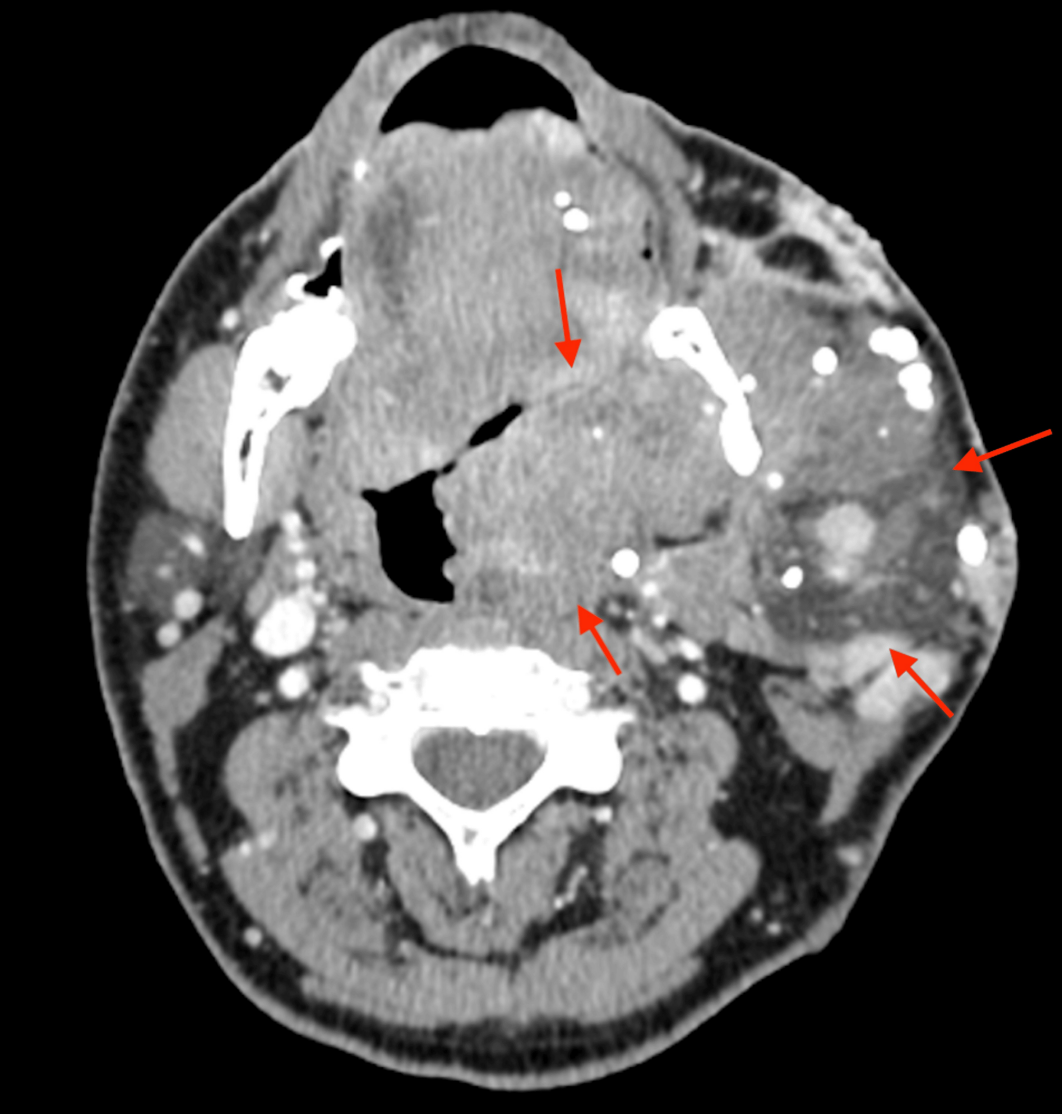
Axial view of a CT scan of a neck at the C2 level of the oropharynx depicting a large left-sided haemangioma extending deep in to the airway indicated by the red arrows CT: Computed tomography

**Figure 3:**
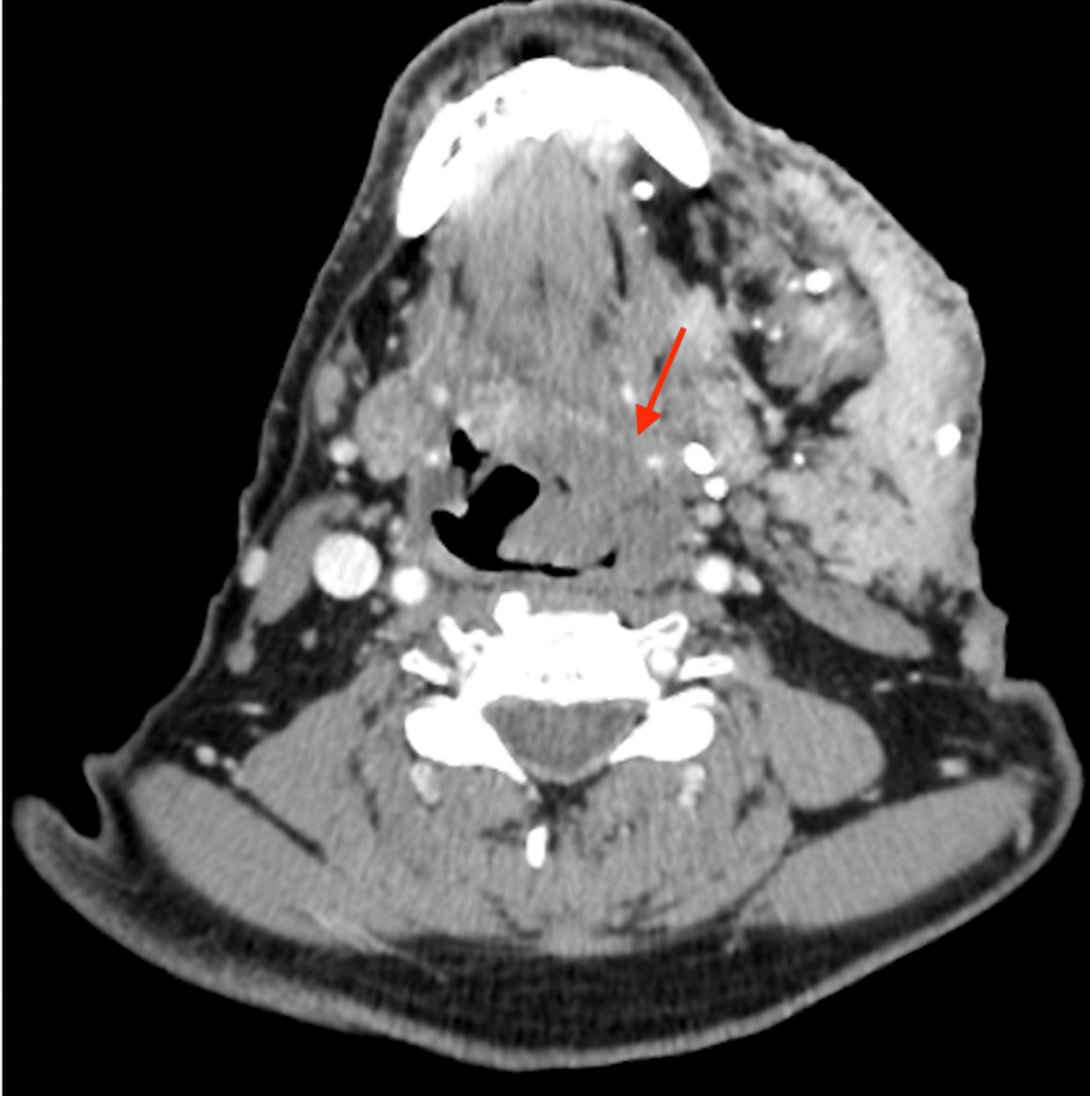
Axial view of a CT scan of the neck at the C3 level of the oropharynx depicting the significant extension of the haemangioma crossing the midline of the airway, as shown by the red arrow CT: Computed tomography

**Figure 4:**
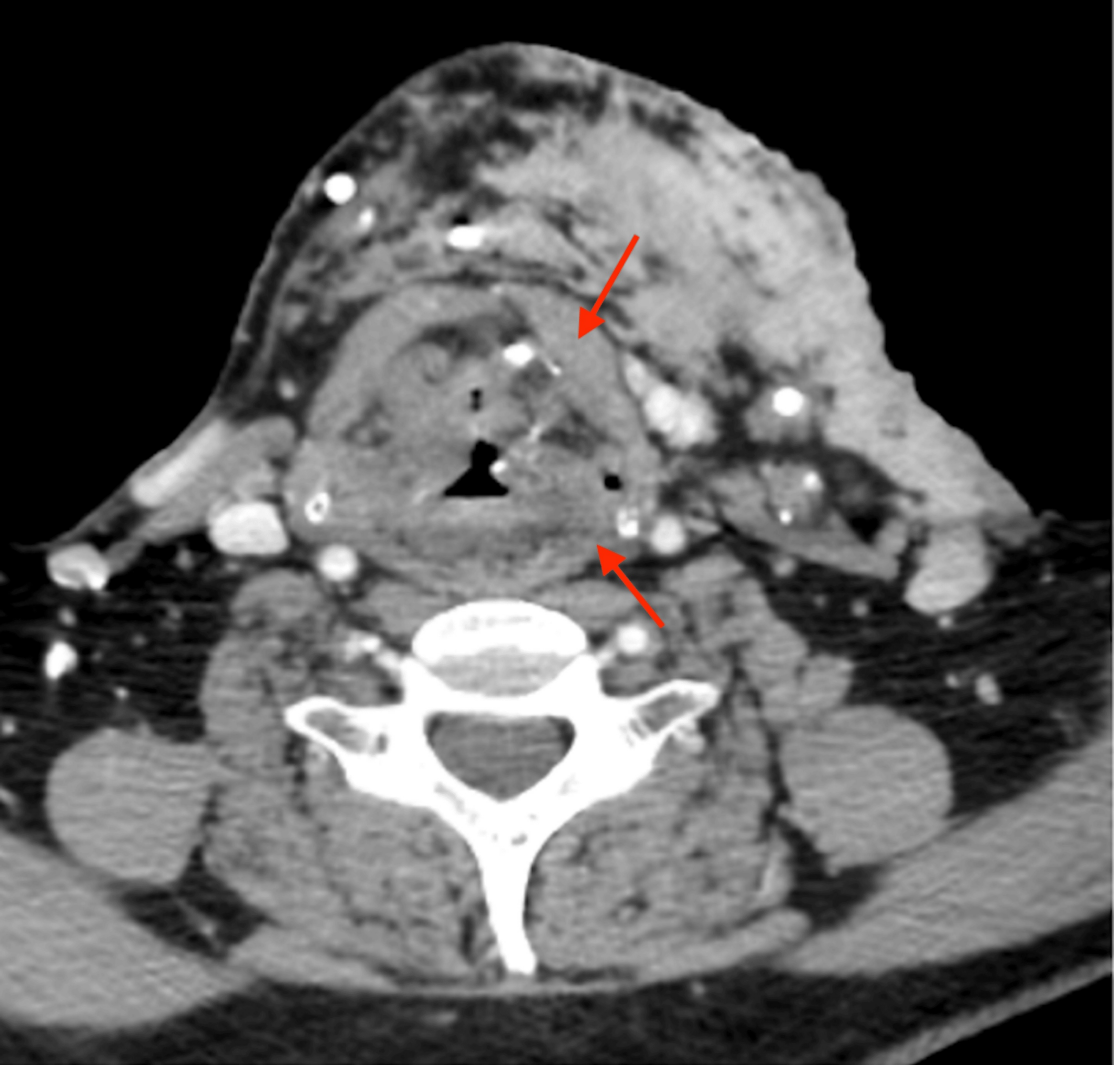
Axial view of a CT scan of the neck at the level of the larynx further delineating extension, as shown by the red arrows CT: Computed tomography

**Figure 5:**
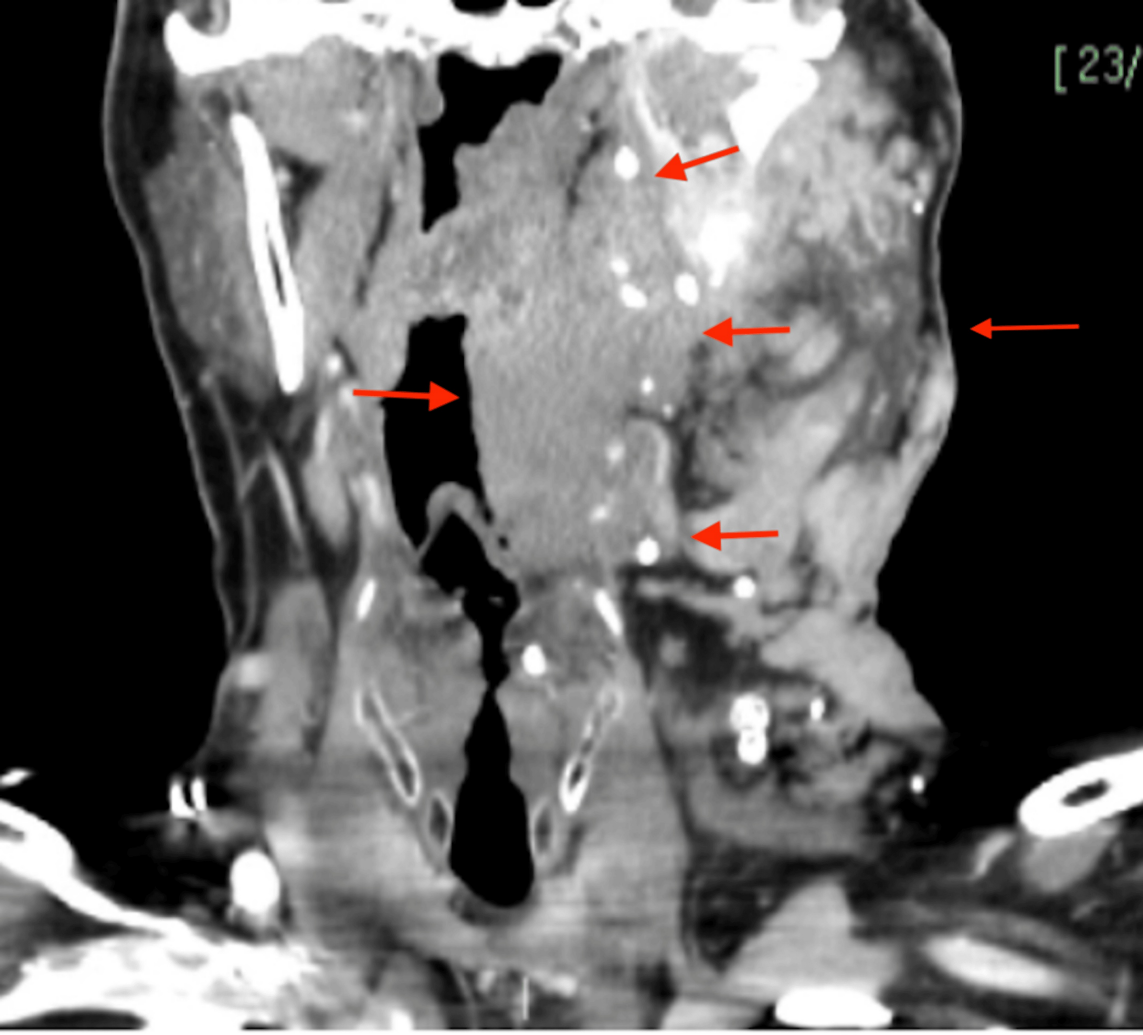
Coronal view of a CT scan of the neck at the level of the epiglottis demonstrating inferior and medial extension, as shown by the red arrows CT: Computed tomography

His blood laboratory results, including his haemoglobin, were all normal. A computed tomography (CT) scan of the neck with contrast was performed to further delineate the true extent of the lesion (see Figures 2-5).

Given the absence of aerodigestive symptoms, a watchful waiting approach was adopted for this patient. He reported a symptom of neck discomfort that was described as mild and occurred only following intense physical activity. This was considered tolerable when weighed against the potential risks associated with medical or surgical intervention. It is well documented that haemangiomas can swell due to increased blood flow following exercise, which was explained to the patient as the most likely cause of his discomfort [4].

A review of the patient's head and neck history also provided clarification regarding concerns about his anaesthetic risk, which had arisen following a difficult general anaesthetic in childhood. At that time, he was advised never to undergo general anaesthesia again. However, advancements in anaesthetic techniques, including awake fibre-optic intubation, mean that his condition should no longer pose a significant risk if the anaesthetic is meticulously planned [5]. This information has been recorded as an alert in his electronic medical record.

It is noteworthy that the patient reports not having been offered any treatment during childhood, having been informed at the time that no therapeutic options were available.

The patient has been made aware of the rare but potential airway complications that could arise due to bleeding or excessive swelling, which may necessitate emergency front-of-neck access should his airway become compromised and intubation prove impossible.

He has agreed to an open follow-up arrangement and has been provided with guidance on recognising warning signs that would require further review in the head and neck clinic.

## Discussion

Haemangiomas with airway compromise are rare, as only a small minority persist into adulthood. These benign vascular tumours can be classified as either congenital or infantile. Congenital haemangiomas, which are fully developed at birth following in utero proliferation, differ from infantile haemangiomas that typically emerge postnatally, exhibit rapid growth during infancy, and subsequently undergo spontaneous involution [1]. Although uncommon overall, a significant proportion of these lesions occur in the head and neck region, with clinical presentations varying depending on size, anatomical location, and associated complications. These range from minor, asymptomatic lesions to large, disfiguring masses that may lead to bleeding, infection, ulceration, organ dysfunction, or airway compromise [1,6,7].

Several studies have emphasised the high prevalence of congenital haemangiomas in the head and neck region. A retrospective analysis by Mulliken et al. reported that approximately 33% of congenital haemangiomas occur in this anatomical area, highlighting the importance of maintaining a high index of suspicion when evaluating vascular anomalies [8]. While congenital haemangiomas generally exhibit a more predictable clinical course than infantile haemangiomas, those affecting the airway pose significant diagnostic and therapeutic challenges, often requiring a multidisciplinary approach for optimal management [9].

The pathophysiology of haemangiomas remains incompletely understood, with various theories implicating genetic predisposition, hypoxic influences, placental origins, hormonal effects, and extrinsic factors, such as maternal smoking and prematurity. North et al. [10] proposed that congenital haemangiomas exhibit distinct vascular characteristics compared with infantile haemangiomas, with evidence suggesting a lack of GLUT-1 expression in congenital subtypes - a key distinguishing histopathological feature. Accordingly, accurate classification and early diagnosis are crucial in differentiating haemangioma subtypes and excluding other potentially malignant conditions, particularly in cases of airway involvement where a delayed diagnosis may have significant consequences [10,11].

Management strategies for haemangiomas have advanced significantly over the past two decades, with pharmacotherapy remaining the cornerstone of treatment for infantile haemangiomas. Propranolol, a non-selective β-blocker, has shown remarkable efficacy in reducing lesion size and associated complications, with a large meta-analysis of 41 studies reporting response rates between 82% and 100%. However, its effectiveness in congenital haemangiomas is considerably lower, as these lesions do not undergo the proliferative phase that β-blocker therapy targets [12]. Corticosteroids, historically the mainstay of treatment before the introduction of propranolol, are now typically reserved for refractory cases requiring symptomatic control. In cases of airway compromise, surgical intervention becomes necessary when medical management is inadequate. Partial or complete excision, often preceded by intralesional corticosteroid injections to reduce lesion vascularity, is a recognised approach for severe cases. Advances in surgical techniques, including endoscopic approaches with CO₂ laser ablation, have facilitated less invasive management of airway-involving haemangiomas, with some studies reporting improved outcomes compared with open excision in select cases [2,7,13]. Postoperative medical therapy is commonly employed to minimise recurrence risk, particularly in patients with incompletely excised lesions.

This case highlights the complexities associated with congenital haemangiomas involving the airway, particularly in adult patients where such presentations are exceedingly rare. The case underscores the importance of early identification, multidisciplinary assessment, and an individualised treatment approach to optimise patient outcomes. Future research should focus on refining classification criteria, further elucidating the pathophysiology of these lesions, and evaluating long-term treatment outcomes to improve clinical management strategies.

## Conclusions

This case report demonstrates that congenital haemangiomas, typically managed conservatively in infancy, can persist into adulthood and become symptomatic. In this 57-year-old patient, mild neck discomfort and minor bleeding during exercise were noted, with flexible nasopharyngolaryngoscopy confirming lesion extension into the airway. Despite these findings, the absence of significant aerodigestive compromise justified a conservative 'watch and wait' approach. This case underscores the variability in clinical presentation and highlights the importance of accurate classification, early diagnosis, and a multidisciplinary management strategy - including pharmacotherapy, surgical intervention when necessary, and advanced anaesthetic techniques - to optimise outcomes and manage potential complications.
